# Supplementary material for: Patients with Cardiovascular Implantable Electronic Devices in the Era of COVID-19 and Their Response to Telemedical Solutions
Source: Medicina (Kaunas). 2022 Jan 21;58(2):160. doi: 10.3390/medicina58020160 (PMC8877859; doi:10.3390/medicina58020160)
Supplement: Supplementary file 1 [file medicina-58-00160-s001.zip › medicina-1525530-supplementary/Questionnaire S1.pdf]

Assessment of telemedicine in COVID-19 survey

1. Please indicate your gender:
    a. Female
    b. Male
2. Please indicate your age:
    a. >80 years
    b. 71-80 years
    c. 61-70 years
    d. 51-60 years
    e. 40-50 years
    f. 31-40 years
    g. <30 years
3. Please indicate your place of residence:
    a. City >1 million residents
    b. City >100,000 residents <1 million residents
    c. City <100,000 residents
    d. Village
4. What cardiac implantable electronic device do you have implanted?
    a. Cardiac pacemaker (PM)
    b. Implantable cardioverter defibrillator (ICD)
    c. Cardiac resynchronization therapy pacemaker/cardioverter-defibrillator (CRT-P/CRT-D)
    d. Other (for example implantable loop recorder, **ILR)**
5. Have you had regular device ambulatory follow-ups since 11/03/2020 (start of restrictions on outpatient clinics/hospitals)?
    a. Yes
    b. No
6. Has the date of the device follow-up been postponed due to the COVID-19 pandemic?
    a. Yes
    b. No
7. If yes, how long was the visit postponed for?
    a. Up to 2 weeks
    b. Up to one month
    c. Up to 3 months
    d. Up to 6 months
    e. Indefinite period
    f. My visit has not been postponed
8. Did you have a teleconsultation about your general health and the implanted device?
    a. Yes
    b. No
9. If yes, are you satisfied with your teleconsultation?
    a. Yes
    b. No
    c. I did not have a teleconsultation
10. Are you satisfied with this way of contact with your doctor?
    a. Yes

    b. No

11. Would you like teleconsultation with a doctor to be more commonly available?
    a. Yes
    b. No
12. Is your device controlled by remote monitoring system?
    a. Yes
    b. No
13. If you answered “No” to the previous question, would you like your device to be controlled by remote monitoring system?
    a. Yes
    b. No
    c. I already have my device controlled by remote monitoring system
14. Would you be able to pay the additional fee for remote monitoring system?
    a. Yes
    b. No
15. If you answered “Yes” to the previous question, what costs per month would you be able to cover?
    a. Up to 20 PLN
    b. Up to 50 PLN
    c. Up to 100 PLN
    d. Up to 200 PLN
    e. Up to 500 PLN
    f. I am not able to cover the costs of telemonitoring
16. During the COVID-19 pandemic, have you ever used the e-prescription?
    a. Yes
    b. No
17. If yes, how would you rate the ability to use such a prescription method on a scale of 0 to 10?

    (0 - worst, 10 - best)
