# Supplementary material for: Patients with Cardiovascular Implantable Electronic Devices in the Era of COVID-19 and Their Response to Telemedical Solutions
Source: Medicina (Kaunas). 2022 Jan 21;58(2):160. doi: 10.3390/medicina58020160 (PMC8877859; doi:10.3390/medicina58020160)
Supplement: Supplementary file 1 [file medicina-58-00160-s001.zip › medicina-1525530-supplementary/Table S1.pdf]

Supplementary Table S1. Detailed results of statistical analyses performed.

	SEX			DEVICE				ACCOMMODATION					AGE					
	Female	Male	p value	Pacemaker	ICD	CRT	p value	City >1 mln	City > 100 000 < 1 mln	City < 100 000	Village	p	<50 yo	51-60 yo	61-70 yo	71-80 yo	>80 yo	p
Regular follow-up visits during the pandemic	52 (54.7)	87 66.4)	0.1006	83 (68.0)	51 (55.4)	5 (45.5)	0.0894	65 (73.9)	18 (51.4)	35 (58.3)	21 (48.8)	0.0158	24 (39.3)	17 (68.0)	32 (68.1)	31 (72.1)	35 (70.0)	0.0015
Delay of rescheduled follow up visit	44 (46.3)	50 (38.2)	0.2757	51 (41.8)	37 (40.2)	5 (45.5)	0.9345	35 (39.8)	15 (42.9)	26 (43.3)	18 (41.9)	0.9744	28 (45.9)	6 (24.0)	17 (36.2)	19 (44.2)	24 (48.0)	0.2689
Satisfaction with teleconsultation when with no previous visit	41 (43.2)	48 (36.6)	0.3943	49 (40.2)	36 (39.1)	4 (36.4)	0.9643	32 (36.4)	14 (40.0)	24 (40.0)	19 (44.2)	0.8574	16 (26.2)	12 (48.0)	17 (36.2)	27 (62.8)	17 (34.0)	0.0032
Satisfaction with the performed teleconsultation	41 (100.0)	47 (97.9)	0.9467	49 (100.0)	35 (97.2)	4 (100.0)	0.9867	32 (100.0)	14 (100.0)	23 (95.8)	19 (100.0)	0.9976	15 (100.0)	12 (100.0)	17 (100.0)	26 (96.3)	17 (100.0)	0.9867
General satisfaction with the interaction with the physician during the teleconsultation for patients with previous in-clinic visit	58 (61.1)	88 (67.2)	0.4184	81 (66.4)	60 (65.2)	5 (45.5)	0.3774	62 (70.5)	21 (60.0)	37 (61.7)	26 (60.5)	0.5339	31 (50.8)	15 (60.0)	31 (66.0)	33 (76.7)	36 (72.0)	0.0539
Want universal teleconsultations regardless of epidemiological restrictions	77 (81.1)	99 (75.6)	0.4137	93 (76.2)	77 (83.7)	5 (45.5)	0.013	69 (78.4)	26 (74.3)	48 (80.0)	33 (76.7)	0.9264	51 (83.6)	18 (72.0)	36 (76.6)	34 (79.1)	37 (74.0)	0.7027
Patients monitored by telemonitoring	12 (12.6)	12 (9.2)	0.537	9 (7.4)	14 (15.2)	1 (9.1)	0.1814	6 (6.8)	3 (8.6)	10 (16.7)	5 (11.6)	0.2779	9 (14.8)	2 (8.0)	7 (14.9)	4 (9.3)	2 (4.0)	0.3342
Patients who want to be monitored by telemonitoring	54 (56.8)	81 (61.8)	0.6976	67 (54.9)	60 (65.2)	8 (72.7)	0.0367	51 (58.0)	21 (60.0)	40 (66.7)	23 (53.5)	0.2291	41 (67.2)	18 (72.0)	24 (51.1)	24 (55.8)	28 (56.0)	0.1121
The possibility of paying additional cost from telemonitoring	32 (33.7)	46 (35.1)	0.935	39 (32.0)	35 (38.0)	4 (36.4)	0.6474	36 (40.9)	11 (31.4)	19 (31.7)	12 (27.9)	0.4529	28 (45.9)	6 (24.0)	15 (31.9)	16 (37.2)	13 (26.0)	0.1583
Acceptable monthly fee for	59.44±50.03	63.08±53.45	0.804	68.54±60.2	54.4±43.5	67.5±39.5	0.3547	71.11±53.76	55±48.22	40.77±26.06	85.83±74.28	0.2416	67.09±57.46	37±26.27	66.88±50.56	53.89±43.81	71.54±62.96	0.4256

Supplementary Table S1. Detailed results of statistical analyses performed.

telemonitoring service																	
Use of the e-prescription system during a pandemic	87 (91.6)	116 (88.5)	0.6026	111 (91.0)	80 (87.0)	11 (100.0)	0.3257	84 (95.5)	28 (80.0)	56 (93.3)	35 (81.4)	0.0123	49 (80.3)	23 (92.0)	44 (93.6)	41 (95.3)	46 (92.0)	0.0725
Patient rating of the e-prescription system (scale 0-10)	8.86±1.86	8.4±2.14	0.0314	8.39±2.29	8.88±1.6	8.45±2.1	0.3618	8.45±2.02	9.14±1.33	8.37±2.24	8.77±2.15	0.2453	8.69±2.01	9.2±1.44	8.74±2.0	8.47±2.3	8.16±2.02	0.0304

Data are presented as number (percentage) of patients or mean ±standard deviation

Statistical analysis

- 10-11 questions – chi-square test
- 12 question - tau-kendalla test

Abbreviations: yo – years old
